# Epidemiology of Developmental Dysplasia of the Hip: Analysis of Japanese National Database

**DOI:** 10.2188/jea.JE20210074

**Published:** 2023-04-05

**Authors:** Hiroki Den, Junichi Ito, Akatsuki Kokaze

**Affiliations:** 1 Department of Hygiene, Public Health, and Preventative Medicine, Showa University School of Medicine, Tokyo, Japan; 2 Department of Orthopaedic Surgery, National Rehabilitation Center for Children with Disabilities, Tokyo, Japan

**Keywords:** developmental dysplasia of the hip, DDH, epidemiology, national database, incidence rate

## Abstract

**Background:**

Developmental dysplasia of the hip (DDH) is a cluster of hip development disorders that affects infants. The incidence of DDH-related dislocation (DDH-dislocation) is reportedly 0.1–0.3%; however, the nationwide incidence of DDH-dislocation in Japan has not been previously reported. The primary aim of this study was to report the nationwide incidence of DDH-dislocation in Japan using the National Database of Health Insurance Claims and Specific Health Checkups of Japan (NDB), and to examine its regional variation across Japan.

**Methods:**

This was a retrospective birth cohort study using the NDB. Data on patients born between 2011 and 2013 and assigned DDH-dislocation-related disease codes during 2011–2018 were extracted. Among these, patients who underwent treatment for DDH-dislocation between 2011 and 2018 were defined as patients with DDH-dislocation.

**Results:**

Across the 2011, 2012, and 2013 birth cohorts, 2,367 patients were diagnosed with DDH-dislocation, yielding the nationwide incidence of 0.076%. Region-specific incidence rates were almost similar across Japan. Secondary analyses revealed that 273 (11.5%) patients were diagnosed at the age of ≥1 year. The effect of birth during the cold months on the incidence of DDH-dislocation was significant (relative risk [RR] = 1.89, 95% confidence interval [CI]: 1.75–2.06). The risk of DDH-dislocation among girls was approximately seven times higher than that among boys.

**Conclusion:**

This is the first study to report the nationwide incidence of DDH-dislocation in Japan, which was estimated at 0.076%. The regional variation was trivial and unlikely to be clinically significant. Thus, the incidence rates were approximately equal across all regions in Japan.

## INTRODUCTION

Developmental dysplasia of the hip (DDH) is a cluster of hip development disorders, which includes dislocation, subluxation, and acetabular dysplasia. DDH is one of the most common hip diseases in infants. Dislocation is defined as the complete displacement of a hip joint, and the natural history of residual dislocation related to DDH is associated with pain and severe osteoarthritis of the hip during young adulthood.^1^^,^^2^ Early detection and treatment of dislocation related to DDH (DDH-dislocation) are highly effective, with >80% success rates.^3^^–^^6^ However, treatment outcomes in patients diagnosed with DDH-dislocation at the age of ≥1 year vary,^7^^,^^8^ suggesting that early detection and treatment are essential for good outcomes in patients with DDH-dislocation. In the 1960s and 1970s, the incidence of DDH-dislocation in Japan was estimated at 1–3%. The subsequent prevention campaigns conducted since 1975 resulted in a decrease in the incidence of DDH-dislocation to 0.1–0.3%^9^; recent studies have reported estimates similar to those or lower.^10^^–^^12^ Nevertheless, these previous estimates were reported on a city or prefecture level, and the nationwide incidence rate of DDH-dislocation in Japan remains unclear.

As Japan stretches in the north-south direction, local incidence rates are likely to vary between regions, making any single estimate unlikely to represent the nationwide incidence. In addition, previous studies have suggested that girls and individuals born during the cold months are more likely to develop DDH than their counterparts^7^^,^^13^^–^^15^; however, the impact of these risk factors may be heterogeneous across the country. Estimating the nationwide incidence and understanding its regional variation are crucial for developing and implementing early detection strategies.

The National Database of Health Insurance Claims and Specific Health Checkups of Japan (NDB) was developed by the Ministry of Health, Labour and Welfare in 2008; it was made available for research purposes in 2013.^16^^–^^19^ Japan has a universal health care system that covers the whole population; the NDB stores >90% of all medical claims. As there is no DDH registry in Japan, using the NDB may be the only feasible way of keeping track of the number of patients with DDH-dislocation and obtaining their demographic and geographic characteristics at the time of diagnosis. The aim of this study was to estimate the nationwide incidence of DDH-dislocation using the NDB data, and to examine its regional variation across Japan. The secondary aim of this study was to estimate the rate of late DDH-dislocation diagnosis, and the effect of sex and birth during the cold months on the incidence of DDH-dislocation per region of Japan.

## METHODS

This was a retrospective birth cohort study based on the NDB. The use of DDH-dislocation patients’ claims stored in the NDB was approved by the Ministry of Health, Labour and Welfare in March 2020 (No. 0298). This study was approved by the ethics committee of the Showa University School of Medicine in January 2020 (No. 3013).

**Figure 1.**
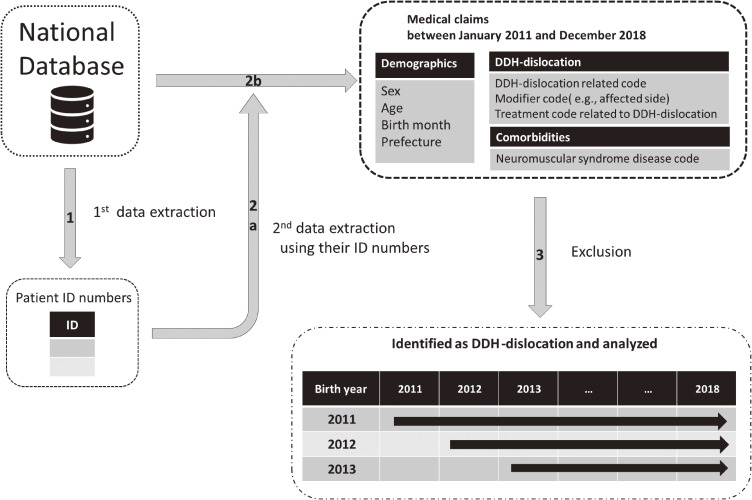
Study design diagram 1st data extraction: Extracted patient ID numbers who were born from January 2011 through December 2013 and assigned DDH-dislocation related codes between January 2011 and December 2018. 2nd data extraction: Extracted medical claims of these patients using their patients ID numbers. Exclusion: Excluded the patients with teratologic, traumatic, and other causes of hip dislocation, and patients who did not receive any treatment for DDH-dislocation between January 2011 and December 2018. DDH, developmental dysplasia of the hip.

In the NDB, each patient has two unique ID numbers (ID1 and ID2). ID1 is generated by encrypting the patient’s health insurance card number, date of birth, and sex. ID2 is generated by encrypting the patient’s name, date of birth, and sex. A two-stage extraction method was used to obtain the claims of the patients with DDH-dislocation (Figure 1). First, we identified both ID1 and ID2 of patients born between 2011 and 2013 who were assigned DDH-dislocation-related codes at any time from January 2011 through December 2018. Second, using their ID numbers, all medical claims of these patients were extracted, including the data on patient sex, age, birth month, prefecture, modifier codes for DDH-dislocation-related codes (used to supplement information about the disease), comorbidities, and any treatment related to DDH-dislocation administered from January 2011 through December 2018.

Medical claims that were assigned the same ID1 were considered as the claims for the same patient. In addition, ID1 might be changed when the patient’s health insurance card number was changed. Therefore, medical claims that were assigned different ID1s but satisfied the following conditions, were also considered as the claims for the same patient: 1) claims were assigned the same ID2; and 2) different ID1s did not overlap during the same period.

DDH-dislocation was identified based on the Japanese standardized disease codes. The DDH-dislocation-related disease names, codes, and their corresponding International Classification of Diseases, 10^th^ revision (ICD-10) codes comprised: congenital dislocation of hip, unilateral (Japanese standardized disease code: 8830552, ICD-10: Q650); congenital dislocation of hip, bilateral (8841054, ICD-10: Q651); congenital dislocation of hip, unspecified (7543010, 7543011, 7543012, ICD-10: Q652); other congenital dislocation of hip (7543007, 8849548, ICD-10: Q658); congenital deformity of hip, unspecified (7543009, ICD-10: Q659), and hip dislocation (8350004, ICD-10: S730).

To exclude teratologic hip dislocations, patients who were assigned neuromuscular syndrome codes (eTable 1) simultaneously or before the DDH-dislocation code assignment were excluded. Further, if the modifier codes for DDH-dislocation described traumatic or other causes for hip dislocation (eTable 2), these patients were excluded from this study as well. Subsequently, patients that did not receive any treatments for DDH-dislocation during the follow-up period were also excluded, as wait-and-watch is not a valid approach to DDH-dislocation.

The possible treatments for DDH-dislocation are shown in eTable 3. Finally, the 2011, 2012, and 2013 birth cohorts were followed up for 7, 6, and 5 years, respectively, and the patients who newly developed DDH-dislocation during these particular time periods were identified.

**Figure 2.**
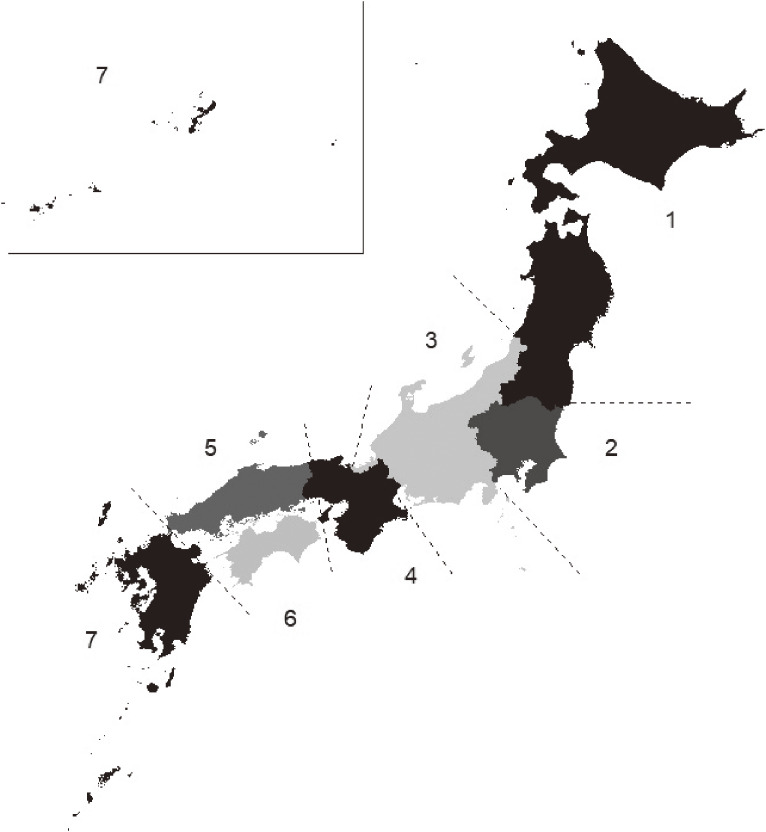
Regions of Japan, including the following prefectures: Region 1 Hokkaido and Tohoku: Hokkaido, Aomori, Iwate, Miyagi, Akita, Yamagata, and Fukushima. Region 2 Kanto: Ibaraki, Tochigi, Gunma, Saitama, Chiba, Tokyo, and Kanagawa. Region 3 Chubu: Niigata, Toyama Ishikawa, Fukui, Yamanashi, Nagano, Gifu, Shizuoka, and Aichi. Region 4 Kinki: Mie, Shiga, Kyoto, Osaka, Hyougo, Nara, and Wakayama. Region 5 Chugoku: Tottori, Shimane, Okayama, Hiroshima, and Yamaguchi. Region 6 Shikoku: Tokushima, Kagawa, Ehime, and Kochi. Region 7 Kyushu and Okinawa: Fukuoka, Saga, Nagasaki, Kumamoto, Oita, Miyazaki, Kagoshima, and Okinawa.

The prefectures where patients were diagnosed with DDH-dislocation for the first time were considered their places of birth. To calculate the incidence rate, the nationwide and prefecture-level birth counts were obtained from e-Stat, a site for Japanese government statistics.^20^ The cases in which the patient’s age at the time of the first DDH-dislocation diagnosis was ≥1 year were considered late diagnoses. The NDB guidelines stipulate that when the number of cases is <10, the estimates cannot be reported to protect the individuals involved. To follow this guideline, Japanese prefectures were divided into seven regions based on an established convention (Figure 2); estimates were reported for these regions. The relative risk (RR) estimates associated with sex (the female effect) and birth during the cold months (November–February) were calculated per region. To estimate the female effect on the incidence of DDH, a subgroup analysis was performed in Region 7. Eight prefectures in Region 7 were divided into the two southernmost prefectures (Okinawa and Kagoshima) and other prefectures; subsequently, the corresponding RR estimates were compared. All statistical analyses were performed using R software version 4.0.2 (R Foundation for Statistical Computing, Vienna, Austria). The chi-square test was used to compare the categorical data. When the chi-square test statistic was significant, the standardized residuals were used for a post-hoc analysis. *P*-values obtained in the post-hoc analyses were adjusted using the Bonferroni method. Finally, *P*-values <0.05 were considered indicative of a statistically significant finding.

## RESULTS

**Figure 3.**
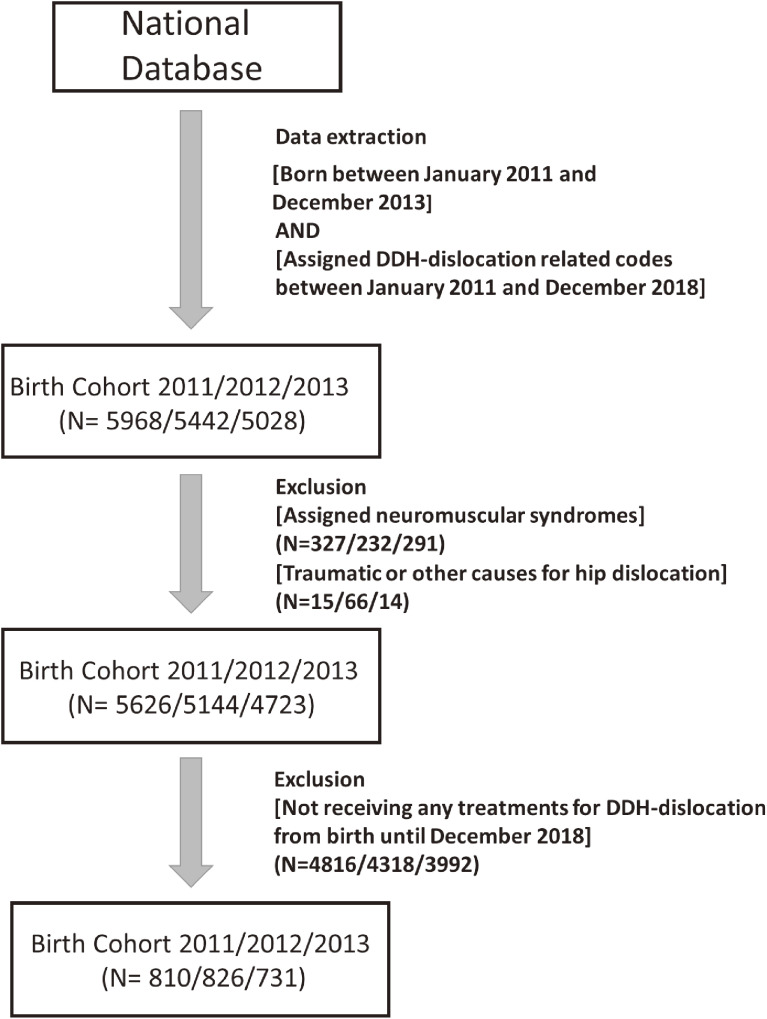
Flowchart of identification of patients with developmental dysplasia of hip-dislocation. DDH, developmental dysplasia of the hip.

**Figure 4.**
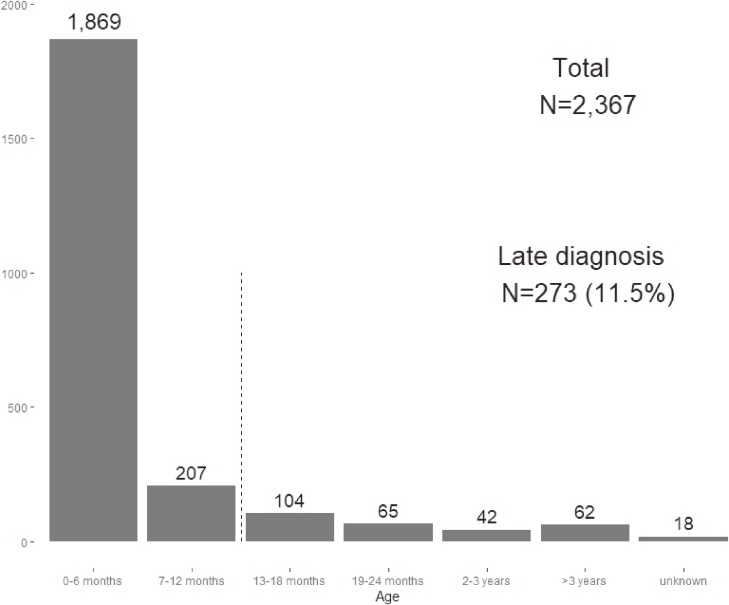
Number of patients with developmental dysplasia of hip-dislocation by age group at diagnosis

By the end of 2018, a total of 810, 826, and 731 patients were identified as having DDH-dislocation in the 2011, 2012, and 2013 birth cohorts, respectively (Figure 3). Overall, the nationwide incidence rate of DDH for the 2011–2013 birth cohorts was 7.59 per 10,000 births (0.076%) (Table 1). In addition, 1,869 (79.0%) patients were diagnosed with DDH-dislocation by the age of 6 months, and 2,076 (87.7%) patients were diagnosed by the age of 1 year. Finally, 273 (11.5%) patients were diagnosed at the age of ≥1 year (late diagnoses), and 62 (2.6%) patients were diagnosed at the age of ≥3 years (Figure 4).

**Table 1.** Nationwide incidence rate of developmental dysplasia of the hip-related dislocation by birth year

Birth Year	Number of Births^a^	Number of patients with DDH-dislocation	Incidence rate (per 10,000)
2011	1,050,684	810	7.71
2012	1,037,164	826	7.96
2013	1,029,762	731	7.10

Total	3,117,610	2,367	7.59

DDH, developmental dysplasia of the hip.

^a^The number of domestic births in Japan.

Table 2 shows the characteristics of patients with DDH-dislocation by birth year. The nationwide incidence rate of DDH-dislocation among those born during the cold months was significantly different from that of patients born during other months (chi-square test, *P* < 0.001).

**Table 2.** Characteristics of patients with developmental dysplasia of hip-dislocation by birth year

Birth year	2011 (*N* = 810)	2012 (*N* = 826)	2013 (*N* = 731)	Total (*N* = 2,367)
Female *N* (%)	697 (86.0)	716 (86.7)	655 (89.6)	2,068 (87.4)

Affected side *N* (%)				
Right	214 (26.4)	212 (25.7)	192 (26.3)	618 (26.1)
Left	460 (56.8)	482 (58.4)	438 (60.0)	1,380 (58.3)
Bilateral	74 (9.1)	66 (8.0)	48 (6.6)	188 (7.9)
Unknown	62 (7.7)	66 (8.0)	53 (7.3)	181 (7.6)

Born in cold months^a^ *N* (%)	374 (46.2)	384 (46.5)	362 (49.5)	1,120 (47.3)
Incidence rate (/10,000 births)				
Born in cold months^a^	11.1	15.2	10.9	11.1
other months	6.1	6.3	5.3	5.9

^a^Cold months include November, December, January, and February.

Region-specific incidence rates are presented in Table 3. The incidence rates were not statistically homogenous (chi-square test, *P* < 0.001). Post-hoc analyses for the chi-square test revealed that the number of patients with DDH-dislocation were significantly higher in Regions 1 and 5 compared with the expected values (*P*-values for standardized residuals of patients with DDH-dislocation; Region 1: *P* = 0.006, Region 5: *P* < 0.001). Table 3 also presents the number of late diagnoses per region; the rates of late diagnoses were similar across the regions (chi-square test, *P* = 0.99).

**Table 3.** Incidence rates and the number of late diagnoses per region for the 2011–13 birth cohorts

Region	Incidence rate (per 10,000 births)				Late diagnosis
	2011	2012	2013	total	*N* (%)

1.Hokkaido and Tohoku	9.20	10.54	7.95	9.23^*^	31 (10.7)
2.Kanto	7.50	6.63	6.42	6.85	82 (11.6)
3.Chubu	6.97	7.62	6.52	7.04	47 (12.4)
4.Kinki	7.54	7.92	7.23	7.57	47 (11.2)
5.Chugoku	10.81	11.72	10.17	10.90^**^	31 (11.5)^***^
6.Shikoku	6.49	7.92	7.07	7.16	
7.Kyushu and Okinawa	7.09	8.16	7.35	7.53	35 (11.7)

Total	7.71	7.96	7.10	7.59	273 (11.5)

Post-hoc chi-square test (*P*-values for the standardized residuals of patients with developmental dysplasia of the hip-dislocation): Region 1^*^: *P* = 0.006, Region 5^**^: *P* < 0.001.

^***^Data for Regions 5 and 6 were combined to protect patient identity.

**Figure 5.**
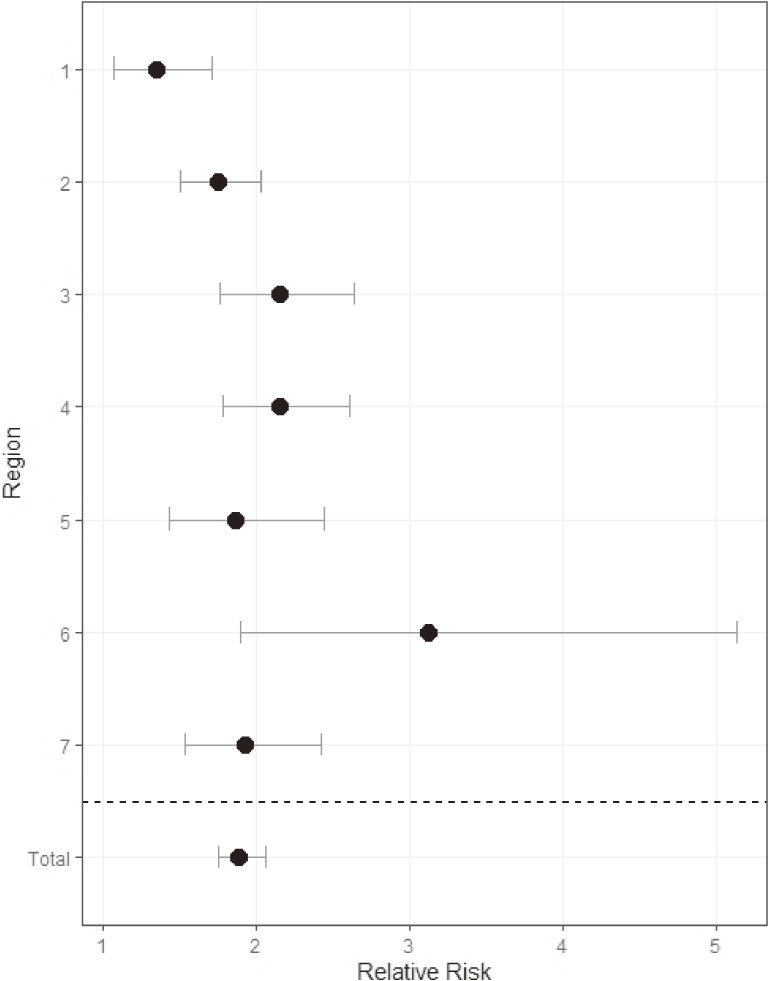
Relative risk estimates of developmental dysplasia of hip-related dislocation in patients born during the cold months (November–February) per region, including 95% confidence intervals.

Birth during the cold months had a significant effect on the incidence of DDH-dislocation (RR 1.89; 95% confidence interval [CI], 1.75–2.06), which was similar across the regions (Figure 5).

**Figure 6.**
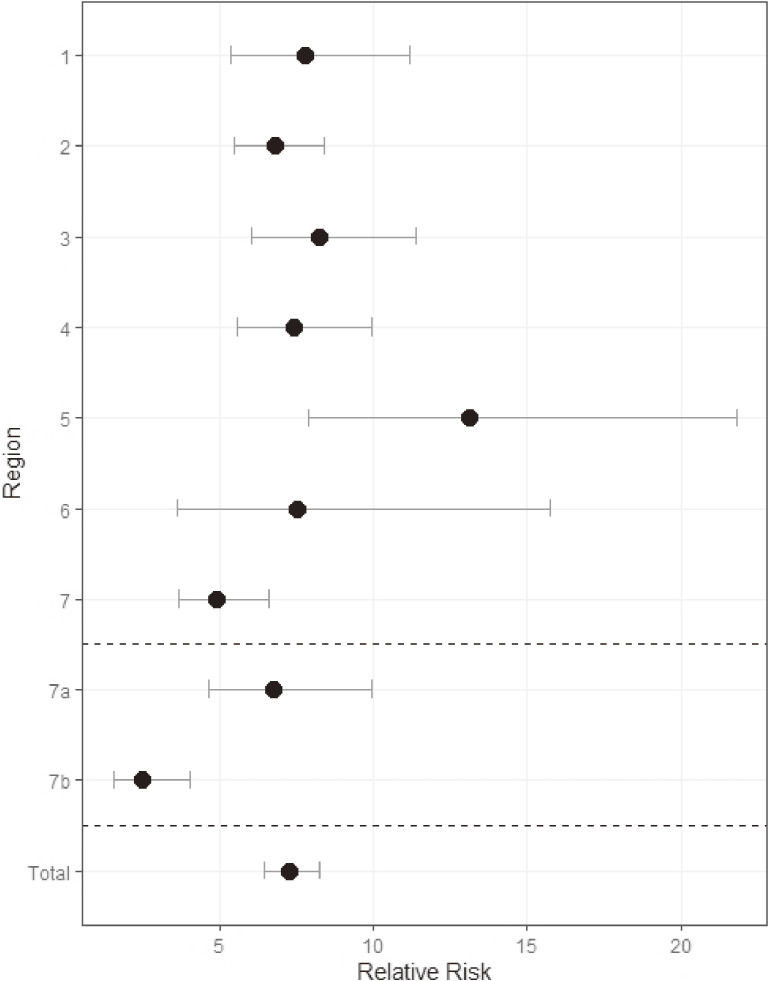
Relative risk estimates of developmental dysplasia of hip-related dislocation in female patients compared to male patients per region, including 95% confidence intervals. 7a includes Fukuoka, Saga, Nagasaki, Kumamoto, Oita, and Miyazaki; 7b includes Kagoshima and Okinawa.

The overall female effect on the incidence of DDH-dislocation was also significant (RR 7.27; 95% CI, 6.44–8.21). Since the female effect observed in Region 7 was smaller than that in the other regions, a subgroup analysis was performed. The RR estimate for the two southernmost prefectures was 2.47 (95% CI, 1.52–4.02), and that for the other prefectures was 6.76 (95% CI, 4.60–9.93), respectively (Figure 6). There was no evidence of interaction between birth during the cold months and the female effect.

## DISCUSSION

To the best of our knowledge, this is the first study to report the nationwide incidence rate of DDH-dislocation in Japan, which was estimated at 0.076% for the birth cohorts during 2011–2013. This rate was lower than that previously reported.^9^ This implies that the prevention campaign conducted by the Japanese Pediatric Orthopaedic Association has been effective and has led to a decrease in the incidence rate of DDH-dislocation. The reported DDH incidence rate (including hip subluxation and acetabular dysplasia) in Taiwan for the 2000–2005 birth cohorts was 0.154%,^21^ while the corresponding point prevalence (including hip subluxation and acetabular dysplasia) estimate in Hong Kong during 2005–2010 was 0.087%.^22^ Among the aforementioned findings, the estimate in Taiwan may be considered comparable to that reported in this study. In contrast, the estimate in Hong Kong appears to be lower than that reported in our study, considering the finding in Hong Kong includes hip subluxation and acetabular dysplasia. This difference may have been due to the climatic and/or ethnic difference.

Recent studies in Yamagata Prefecture (Region 1; 2010–2015), Niigata city (Region 3; 2010–2013), and Okinawa Prefecture (Region 7; 2011–2014) have reported a DDH-dislocation incidence of 0.16%,^11^ 0.04%,^10^ and 0.035%, respectively.^12^ These findings are not directly comparable with our findings, as our estimates were obtained on regional levels. However, the aforementioned estimates are similar to our estimates. In addition, the proportion of sex and of affected sides were similar in our study and previous studies based in Japan.^7^^,^^14^ A multi-center study on late diagnoses reported 199 (15%) such cases in Japan,^7^ a finding similar to that in our study (11.4%). These comparisons suggest that our study method has satisfactory validity.

The incidence rates in Regions 1 and 5 were slightly higher than those in other regions. Although these differences were statistically significant, the crude difference between the lowest (Region 2: 6.85) and highest (Region 5: 10.90) incidence rate was 4 in 10,000 births. As the annual number of births in Japan is <900,000, this difference is unlikely to be clinically significant. Overall, the rates of DDH-dislocation appear comparable across Japan. However, these findings may not be generalizable to the prefecture or city level. Nonetheless, our findings may be utilized to estimate the required number of pediatric orthopedic doctors in each region.

The rates of late diagnoses were similar across regions, ranging from 10.7–12.4%. These findings suggest that at least 1 in 10 patients with DDH-dislocation is missed, despite all infants undergoing routine check-ups at the age of 3–6 months. A previous study has shown that most patients with late diagnoses in Japan undergo routine hip checkups that reveal no abnormalities.^7^ Given that the incidence of DDH-dislocation is low and the number of births in Japan is decreasing, orthopedic doctors are unlikely to encounter a patient with DDH-dislocation. In fact, a Japanese study revealed that 32% of orthopedic doctors have not encountered a patient with DDH-dislocation in their career.^23^ Further, some DDH cases are difficult to detect by physical examination alone, and a study in Germany has reported that an ultrasound hip screening system resulted in a decrease in the rate of late presentation.^24^ To reduce the rate of late diagnoses in Japan, an ultrasound hip screening system and an educational system for inexperienced physicians may be required.

Birth during the cold months is a well-known risk factor for DDH-dislocation.^25^ This study supports the hypothesis that infants born during the cold months need to wear a lot of clothes, which disturbs the infants’ hip movements and, in turn, increases the incidence of DDH-dislocation.^25^ In this study, the risk of DDH-dislocation associated with birth during the cold months was about twice as high as that associated with birth during the other months. Further, the RR estimate for Region 1 was lower than that for the other regions, which was possibly because this region corresponds to the northernmost part of Japan, where the cold months are longer than those elsewhere, extending to March and April. Consequently, the RR estimate in Region 1 tended toward the null.

The risk of DDH-dislocation among girls was approximately seven times higher than that among boys. The etiology for the higher incidence of DDH-dislocation among girls remains unclear. Joint laxity in girls due to the influence of female sex hormones is considered as one of the possibilities.^13^^,^^26^ The magnitude of the female effect was similar across Japan, except for the southernmost prefectures (RR 2.47). This difference in risk estimates may result from genetic differences between the populations in Okinawa and those in other prefectures. A previous cluster analysis has reported that the inhabitants of Okinawa Prefecture may be genetically different from those across the rest of Japan; meanwhile, Kagoshima Prefecture is considered to be the closest to Okinawa Prefecture both geographically and genetically.^27^ In addition, a separate study has shown that the Okinawans are prone to unique phenotypes that are distinct from those observed in Japan’s other prefectures.^28^ Further, no genetic affinity has been observed between the Taiwanese and indigenous people in Okinawa Prefecture, despite geographic proximity between Taiwan and Okinawa Prefecture.^29^ In fact, 89% of patients with DDH in Taiwan are female.^30^ Studies in Hong Kong and Singapore have reported female-to-male ratios in DDH as 3:1 or 4:1, respectively, with 63% of DDH patients being female.^13^^,^^22^^,^^31^ These estimates are lower than that observed in our study (87.4%). Loder et al have proposed that there is minimal gender variability based on ethnicity among patients with DDH^13^; however, we assumed that ethnicity affects the gender variability among patients with DDH.

There are several limitations to this study. First, even though the NDB stores >90% of the medical claims, some patients with DDH-dislocation might not have been included. In addition, some patients with DDH-dislocation might have been assigned codes other than those used in this study. Consequently, the incidence rates reported in this study might have been underestimates. Second, some doctors may choose to treat rather than observe patients with DDH-subluxation. Such cases might have been subsequently included as DDH-dislocation, leading to incidence overestimation. Third, the follow-up periods for the birth cohorts in this study were different. Due to the NDB framework, the 2013 birth cohort was followed-up for 2 years less than the 2011 birth cohort; therefore, the incidence of DDH-dislocation in the 2013 birth cohort might have been an underestimate; although the diagnosis of DDH-dislocation is unlikely to occur at the age of ≥5 years. In fact, in the 2011 birth cohort, the final 2 years of the follow-up period yielded less than 10 additional patients. Overall, a 5-year follow-up period is sufficient in this context, as a longer follow-up does not appear to affect the resulting estimates. Finally, data on risk factors, such as family history and breech presentation, are not available in the NDB; thus, these risk factors were not evaluated in this study.^15^ Consequently, the estimated effects of birth during the cold months and the female effect might have been affected by these confounding factors in this study. Despite these limitations, the NDB remains a useful data source for an epidemiological study, allowing the estimation of the nationwide incidence of DDH-dislocation and the associated risk factors of this rare disease.

In summary, this study was the first to estimate the nationwide incidence of DDH-dislocation in Japan using the NDB, which was 0.076%. The rate of late diagnosis was 11.5%, which was similar between the regions. The risk of DDH-dislocation for birth during the cold months was approximately double of that for births during other months. The effect of birth during the cold months was observed throughout Japan. Meanwhile, the risk of DDH-dislocation among girls was approximately seven times higher than that among boys. The female effect was smaller in the two southernmost prefectures compared with elsewhere, which may be accounted for by the genetic differences between the southernmost and other regions in Japan. Further studies are needed to investigate the causes of late diagnosis throughout Japan.

## SUPPLEMENTARY MATERIAL

eTable 1. Neuromuscular syndrome diseases

eTable 2. Modifier codes and their descriptions that were excluded from the developmental dysplasia of hip-dislocation cases

eTable 3. Possible treatments for developmental dysplasia of hip-dislocation

## References

[1] Yang S, Zusman N, Lieberman E, Goldstein RY. Developmental dysplasia of the hip. Pediatrics. 2019;143(1):e20181147. 10.1542/peds.2018-1147 30587534

[2] Ganz R, Leunig M, Leunig-Ganz K, Harris WH. The etiology of osteoarthritis of the hip: an integrated mechanical concept. Clin Orthop Relat Res. 2008;466(2):264–272. 10.1007/s11999-007-0060-z 18196405 PMC2505145

[3] Wada I, Sakuma E, Otsuka T, . The Pavlik harness in the treatment of developmentally dislocated hips: results of Japanese multicenter studies in 1994 and 2008. J Orthop Sci. 2013;18(5):749–753. 10.1007/s00776-013-0432-z 23812768 PMC3778211

[4] Cashman JP, Round J, Taylor G, Clarke NM. The natural history of developmental dysplasia of the hip after early supervised treatment in the Pavlik harness. A prospective, longitudinal follow-up. J Bone Joint Surg Br. 2002;84(3):418–425. 10.1302/0301-620X.84B3.0840418 12002504

[5] Grill F, Bensahel H, Canadell J, Dungl P, Matasovic T, Vizkelety T. The Pavlik harness in the treatment of congenital dislocating hip: report on a multicenter study of the European Paediatric Orthopaedic Society. J Pediatr Orthop. 1988;8(1):1–8. 10.1097/01241398-198801000-00001 3335614

[6] Kotlarsky P, Haber R, Bialik V, Eidelman M. Developmental dysplasia of the hip: what has changed in the last 20 years? World J Orthop. 2015;6(11):886–901. 10.5312/wjo.v6.i11.886 26716085 PMC4686436

[7] Hattori T, Inaba Y, Ichinohe S, . The epidemiology of developmental dysplasia of the hip in Japan: findings from a nationwide multi-center survey. J Orthop Sci. 2017;22(1):121–126. 10.1016/j.jos.2016.08.009 27616132

[8] Thomas SR. A review of long-term outcomes for late presenting developmental hip dysplasia. Bone Joint J. 2015;97-B(6):729–733. 10.1302/0301-620X.97B6.35395 26033050

[9] Yamamuro T, Ishida K. Recent advances in the prevention, early diagnosis, and treatment of congenital dislocation of the hip in Japan. Clin Orthop Relat Res. 1984;(184):34–40. 6705362

[10] Murakami R, Takahashi M, Watanabe K, . Trends in incidence rates of developmental dysplasia of the hip during 1975–2013 in Niga city. J Jpn Ped Orthop Ass. 2017;26(1):1–5 (in Japanese).

[11] Sasaki K, Ishii M, Ida H, . Screening process and delayed diagnosis of developmental dysplasi of the hip in Yamagata prefecture. J Jpn Ped Orthop Ass. 2017;26(1):68–71 (in Japanese).

[12] Kinjyo T, Sugiura Y, Nishi R, . The current status of late diagnosis for DDH and improvement of the secondary screening system in Okinawa prefecture; build a remote control X-ray daignostic system. J Jpn Ped Orthop Ass. 2016;25(2):281–283 (in Japanese).

[13] Loder RT, Skopelja EN. The epidemiology and demographics of hip dysplasia. ISRN Orthop. 2011;2011:238607. 10.5402/2011/238607 24977057 PMC4063216

[14] Satsuma S, Kobayash D, Hamamura S. Epidemiological study of Developmental dysplasia of the hip. J Jpn Ped Orthop Ass. 2008;17(2):298–302 (in Japanese).

[15] Vaquero-Picado A, González-Morán G, Garay EG, Moraleda L. Developmental dysplasia of the hip: update of management. EFORT Open Rev. 2019;4(9):548–556. 10.1302/2058-5241.4.180019 31598333 PMC6771078

[16] Ishimaru M, Matsui H, Ono S, Hagiwara Y, Morita K, Yasunaga H. Preoperative oral care and effect on postoperative complications after major cancer surgery. Br J Surg. 2018;105(12):1688–1696. 10.1002/bjs.10915 30088267

[17] Okumura Y, Sakata N, Takahashi K, Nishi D, Tachimori H. Epidemiology of overdose episodes from the period prior to hospitalization for drug poisoning until discharge in Japan: an exploratory descriptive study using a nationwide claims database. J Epidemiol. 2017;27(8):373–380. 10.1016/j.je.2016.08.010 28242045 PMC5549249

[18] Toyokawa S, Maeda E, Kobayashi Y. Estimation of the number of children with cerebral palsy using nationwide health insurance claims data in Japan. Dev Med Child Neurol. 2017;59(3):317–321. 10.1111/dmcn.13278 27644438

[19] Igari H, Yamagishi K, Yamazaki S, . Epidemiology and treatment outcome of pneumonia: Analysis based on Japan national database. J Infect Chemother. 2020;26(1):58–62. 10.1016/j.jiac.2019.07.001 31353202

[20] Portal Site of Official Statistics of Japan. Accessed Dec 21, 2020. https://www.e-stat.go.jp/.

[21] Chang CH, Chiang YT, Chen L, Kuo KN. The influence of health policy on early diagnosis and surgical incidence of developmental dysplasia of the hip. PLoS One. 2018;13(7):e0200995. 10.1371/journal.pone.0200995 30059550 PMC6066215

[22] Tong SH, Eid MA, Chow W, To MK. Screening for developmental dysplasia of the hip in Hong Kong. J Orthop Surg (Hong Kong). 2011;19(2):200–203. 10.1177/230949901101900214 21857045

[23] Takei S, Ito J, Kosaki K. The diagnostic accuracy of X-ray in patients with dvelopmental dysplasia of the hip among orthopedic surgeons. J Jpn Ped Orthop Ass. 2017;26(2):323–327 (in Japanese).

[24] Wirth T, Stratmann L, Hinrichs F. Evolution of late presenting developmental dysplasia of the hip and associated surgical procedures after 14 years of neonatal ultrasound screening. J Bone Joint Surg Br. 2004;86(4):585–589. 10.1302/0301-620X.86B4.14586 15174558

[25] Loder RT, Shafer C. Seasonal variation in children with developmental dysplasia of the hip. J Child Orthop. 2014;8(1):11–22. 10.1007/s11832-014-0558-3 24500336 PMC3935022

[26] Carter C, Wilkinson J. Persistent Joint Laxity and Congenital Dislocation of the Hip. J Bone Joint Surg Br. 1964;46:40–45. 10.1302/0301-620X.46B1.40 14126235

[27] Watanabe Y, Isshiki M, Ohashi J. Prefecture-level population structure of the Japanese based on SNP genotypes of 11,069 individuals. J Hum Genet. 2021;66(4):431–437. 10.1038/s10038-020-00847-0 33051579

[28] Bendjilali N, Hsueh WC, He Q, . Who are the Okinawans? Ancestry, genome diversity, and implications for the genetic study of human longevity from a geographically isolated population. J Gerontol A Biol Sci Med Sci. 2014;69(12):1474–1484. 10.1093/gerona/glt203 24444611 PMC4271021

[29] Sato T, Nakagome S, Watanabe C, . Genome-wide SNP analysis reveals population structure and demographic history of the ryukyu islanders in the southern part of the Japanese archipelago. Mol Biol Evol. 2014;31(11):2929–2940. 10.1093/molbev/msu230 25086001

[30] Chang CH, Chiang YT, Lee ZL, Kuo KN. Incidence of surgery in developmental dysplasia of the hip in Taiwan. J Formos Med Assoc. 2007;106(6):462–466. 10.1016/S0929-6646(09)60295-3 17588839

[31] Hoaglund FT, Kalamchi A, Poon R, Chow SP, Yau AC. Congenital hip dislocation and dysplasia in Southern Chinese. Int Orthop. 1981;4(4):243–246. 7228460 10.1007/BF00266064

