# Supplementary material for: Epidemiology of Developmental Dysplasia of the Hip: Analysis of Japanese National Database
Source: J Epidemiol. 2023 Apr 5;33(4):186–92. doi: 10.2188/jea.JE20210074 (PMC9939923; doi:10.2188/jea.JE20210074)
Supplement: Supplementary file 1 [file je-33-186-s001.pdf]

eTable 1. Neuromuscular syndrome diseases

Code*	ICD-10 code	ICD-10 Code Description
3314006	G919	Hydrocephalus, unspecified
3439002	G808	Other cerebral palsy
3439006	G80	Cerebral palsy
3439010	G801	Spastic diplegic cerebral palsy
7419003		
7419008	Q059	Spina bifida, unspecified
7419009		
7423007	Q039	Congenital hydrocephalus, unspecified
7423008	Q043	Other reduction deformities of brain
8833885	G808	Other cerebral palsy
8835215	G803	Athetoid cerebral palsy
8835804	Q054	Unspecified spina bifida with hydrocephalus
8835994	T913	Sequelae of injury of spinal cord
8836281	Q743	Arthrogryposis multiplex congenita
8840270	M2435	Pathological dislocation of hip
8840809	Q057	Lumbar spina bifida without hydrocephalus
8842160	G803	Athetoid cerebral palsy
8845811	D177	Benign lipomatous neoplasm of other sites
8846326	G409	Epilepsy, unspecified

ICD-10, International Classification of Diseases, 10th revision.

* Japanese standardized disease code

eTable 2. Modifier codes and their descriptions that were excluded from the developmental dysplasia of hip-dislocation cases

Disease modifier code	Code description
3028	Traumatic
4040	Recurrent
5025	Suppurative
5055	Spastic
5078	Habitual
5182	Paralytic

eTable 3. Possible treatments for developmental dysplasia of hip-dislocation

Code*	Treatment
140043410	Hip spica cast
140043530	Hip spica bivalved cast
140043630	Hip spica plastic cast
140043750	Hip spica plastic bivalved cast
140043850	Hip splint
140045810	Hip spica cast for DDH
140045930	Hip spica bivalved cast for DDH
140046030	Hip spica plastic cast for DDH
140048010	Indirect traction
150027710	osteotomy(femur)
150033910	Closed reduction (hip)
150035050	Pavlik Harness
150035110	Closed reduction for DDH (bilateral)
150035310	Open reduction (hip)
150036110	Open reduction for DDH
150064710	Pelvic osteotomy
150243010	Direct traction
150308910	Proximal femur osteotomy

DDH, Developmental dysplasia of the hip.

* Japanese standardized treatment code
